# Effects of estradiol on in vitro maturation of buffalo and goat oocytes

**DOI:** 10.1002/rmb2.12350

**Published:** 2020-09-25

**Authors:** Hurum Maksura, Narsisa Akon, Md Nuronnabi Islam, Ireen Akter, Avijit Kumar Modak, Asma Khatun, Md Hasanur Alam, Md Abul Hashem, Md Ruhul Amin, Mohammad Moniruzzaman

**Affiliations:** ^1^ Department of Animal Science Bangladesh Agricultural University Mymensingh Bangladesh

**Keywords:** 17β‐estradiol, buffalo, goat, in vitro maturation, oocyte

## Abstract

**Purpose:**

The effects of estradiol on oocyte development seem to be varied among species. The present study investigated the effects of 17β‐estradiol on in vitro maturation of buffalo and goat oocytes.

**Methods:**

Cumulus oocyte complexes (COCs) were aspirated from large antral follicles of slaughtered buffalo and goat ovaries. COCs were cultured in TCM‐199 medium supplemented with 0, 0.5, 1, and 1.5 µg/mL of 17β‐estradiol for in vitro maturation. Then, oocytes were used for the examination of state of nuclear maturation and cumulus expansion.

**Results:**

In both species, oocytes treated with 17β‐estradiol showed higher cumulus expansion rate than control (0 µg/mL treated). In buffalo, the percentage of oocytes matured to the metaphase II (MII) stage increased in the concentration‐dependent manner of 17β‐estradiol. Similarly, estradiol positively influenced nuclear maturation of goat oocytes in vitro.

**Conclusions:**

Estradiol has promoting effects on normalprogress of in vitro oocyte meiosis in buffalos and goats.

## Graphical abstract

The effects of estradiol on oocyte maturation, ovulation, and embryo development seem to be species‐dependent. The present study investigated the effects of 17β‐estradiol on in vitro maturation in buffalo and goat oocytes. The results showed that estradiol enhanced meiotic progression of both buffalo and goat oocytes in vitro.

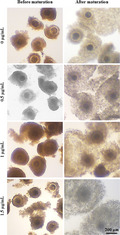

## 1 INTRODUCTION

Assisted reproductive technologies (ARTs) can be applied to improve reproductive efficiencies in livestock species. In addition, ARTs have significant roles in the preservation of endangered species.^1^ ARTs including artificial insemination (AI), cryopreservation, intra‐cytoplasmic sperm injection (ICSI), multiple ovulation and embryo transfer (MOET), and in vitro embryo production (IVEP) have been used for improvement of domestic animals. In buffaloes, the response to superovulation treatment is poor, and ultimately, MOET is hardly applicable. However, oocytes recovered from slaughtered animals are used for IVEP. In vitro maturation (IVM) is the initial step of IVEP technology. IVM of oocytes involves collection of cumulus oocyte complexes (COCs) from antral follicles and culturing them until they reach to the metaphase II (MII) stage.^2^

The success in IVM of oocytes depends on many factors including the size of follicles and oocytes,^3^ the maturation environment,^4^, ^5^ and the culture media used.^6^, ^7^, ^8^ Steroids influence oocyte maturation in vitro.^9^ Estradiol is a major female steroid hormone, produced from cholesterol. To improve the nuclear and cytoplasmic maturation of oocytes as well as the expansion of the cumulus cells, gonadotrophin hormones and estradiol are usually used in IVM of buffalo^10^ and caprine^11^ oocytes. Although the estradiol is supplemented occasionally in IVM, its role in IVM of oocytes remains contentious. Estradiol has been shown to increase IVM of bovine oocytes^12^, ^13^ but Gliedt and colleagues have reported that estradiol negatively regulates cumulus expansion and in vitro embryo production in bovine.^14^ It has also been reported that estradiol reduces the maturation of mouse oocytes^15^, ^16^ and that in vitro maturation of pig oocytes has been inhibited by estradiol.^17^ A high concentration of estradiol (100 µg/mL) has also inhibited oocyte maturation in boer goats.^18^ High concentration of estradiol inhibits spindle formation and polar body extrusion in bovine oocytes.^19^ The effects of estrogen on oocyte maturation, ovulation, and embryo development seem to be species‐dependent.^20^ Its role is still unclear in some mammalian species.

Black Bengal goats are important genetic resource, available in Bangladesh and India. They are dwarf in size and popular for delicious meat with high marbling, high prolificacy, disease resistance, and adaptability in high temperature and humidity. Similarly, buffalo is economically an important species in livestock agriculture in Asia. They are popular for their high butter fat content in their milk, high feed conversion efficiency, and high disease resistance. In Bangladesh, buffaloes are non‐descriptive and river type. A few reports are available on in vitro development of oocytes in Black Bengal goats and indigenous river buffaloes in Bangladesh.

The aim of this study was to examine the effects of estradiol on in vitro maturation of oocytes from river buffaloes and Black Bengal goats.

## 2 MATERIALS AND METHODS

### 2.1 Chemicals

Unless otherwise mentioned, all chemicals were purchased from Sigma‐Aldrich (St. Louis).

### 2.2 Collection and processing of ovaries

Ovaries were collected from indigenous river buffaloes and Black Bengal goats at local slaughter house and kept in a thermo flask containing 0.9% physiological saline for transportation to the laboratory. The ovaries were washed in Dulbecco's phosphate buffer saline (DPBS) solution supplemented with gentamycin (50 µg/mL) once and following three times in DPBS. After trimming surrounding tissues, ovaries were washed again with saline solution.

### 2.3 Collection of COCs

The collection and in vitro maturation of cumulus oocyte complexes (COCs) were done according to Totey et al^21^ with some modifications. Briefly, COCs were aspirated from visible large antral follicles having diameter of 4‐6 mm using a syringe (10 mL; Henke Sass Wolf) and needle (18 G; Henke Sass Wolf). COCs with oocyte of 120‐125 µm in diameter (excluding the zona pellucida) were selected with the help of a stereomicroscope. Diameters of oocytes were measured using an ocular micrometer attached to an inverted microscope (Labomed, Inc). COCs containing healthy oocytes were selected based on their morphological appearance (uniformly granulated cytoplasm surrounded by multilayered compact cumulus cells) for further experimentation.

### 2.4 In vitro maturation of COCs

The maturation medium was prepared with TCM‐199 supplemented with 0.1 mg/mL sodium pyruvate, 0.08 mg/mL gentamycin sulfate, 5% (v/v) fetal bovine serum (FBS), and 100 ng/mL follicle‐stimulating hormone (FSH).^22^ In order to investigate the effects of 17β‐estradiol, maturation medium was also supplemented with 0, 0.5, 1.0, or 1.5 µg/mL of 17β‐estradiol. COCs were placed in 50 µL maturation droplets of maturation medium under paraffin oil. Each droplet was placed in a separate culture dish (No. 430165, 35 mm cell culture dish, Corning Incorporated). They were cultured for 24 hours at 38.5°C temperature with 5% CO_2_ in humidified air.

### 2.5 Assessment of cumulus expansion and meiotic stage of oocytes

After in vitro maturation, the assessment of cumulus expansion was carried out as described by Maruska et al^23^ with some modifications. Briefly, COCs with one or two layers expanded, one‐half of the cumulus expanded, all layers expanded other than last layers of corona radiata, or all layers expanded, including corona radiate, were classified as expanded COCs. All of the COCs other than expanded COCs such as COCs without cumulus expansion (no observable sign of cumulus expansion) were classified as non‐expanded COCs. Cumulus cells surrounding the oocytes were mechanically removed with the help of 0.1% (w/v) hyaluronidase and using a small‐bore pipette. Denudate oocytes were fixed in aceto‐ethanol (acetic acid:ethanol = 1:3) for 48 hours and stained with 1% (w/v) aceto‐orcein to examine the stage of oocyte maturation. The stained oocytes were classified on the basis of morphology of the chromatin and nuclear envelope.^24^, ^25^, ^26^, ^27^ Oocytes after resumption of meiosis, the stages were classified into early diakinesis (ED), late diakinesis (LD), metaphase I (MI) and metaphase II (MII). Oocytes showing cytoplasmic or nuclear abnormalities were considered degenerated oocytes.

### 2.6 Data presentation and statistical analysis

All data from cumulus expansion and meiotic stage assessment experiments were subjected to one‐way ANOVA followed by Tukey's HSD test (IBM SPSS Statistics 22). Differences at *P* < .05 were considered statistically significant.

## 3 RESULTS

### 3.1 Effect of 17β‐estradiol on cumulus expansion during in vitro maturation of COCs

**FIGURE 1:**
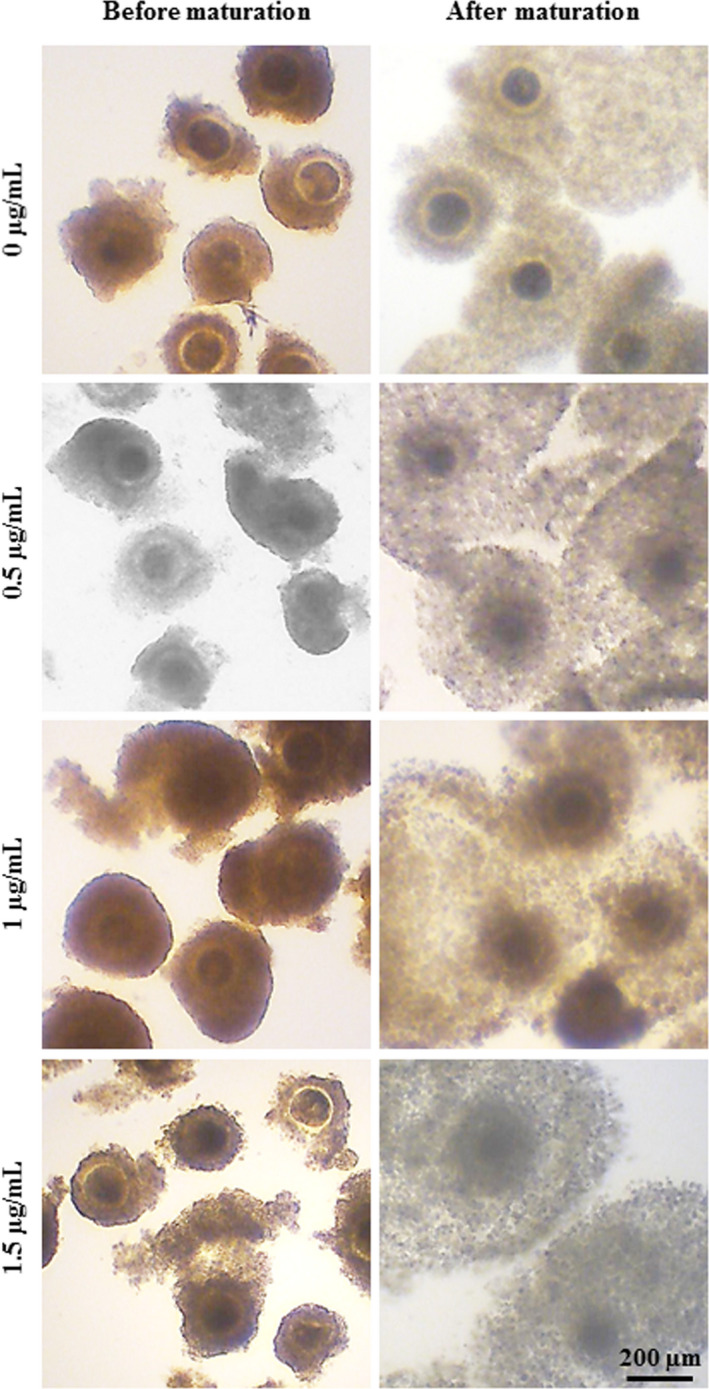
Buffalo cumulus oocyte complexes (COCs) before and after in vitro maturation. The in vitro maturation medium was supplemented with 0, 0.5, 1, and 1.5 µg/mL of 17β‐estradiol. Scale bar represents 200 μm

**FIGURE 2:**
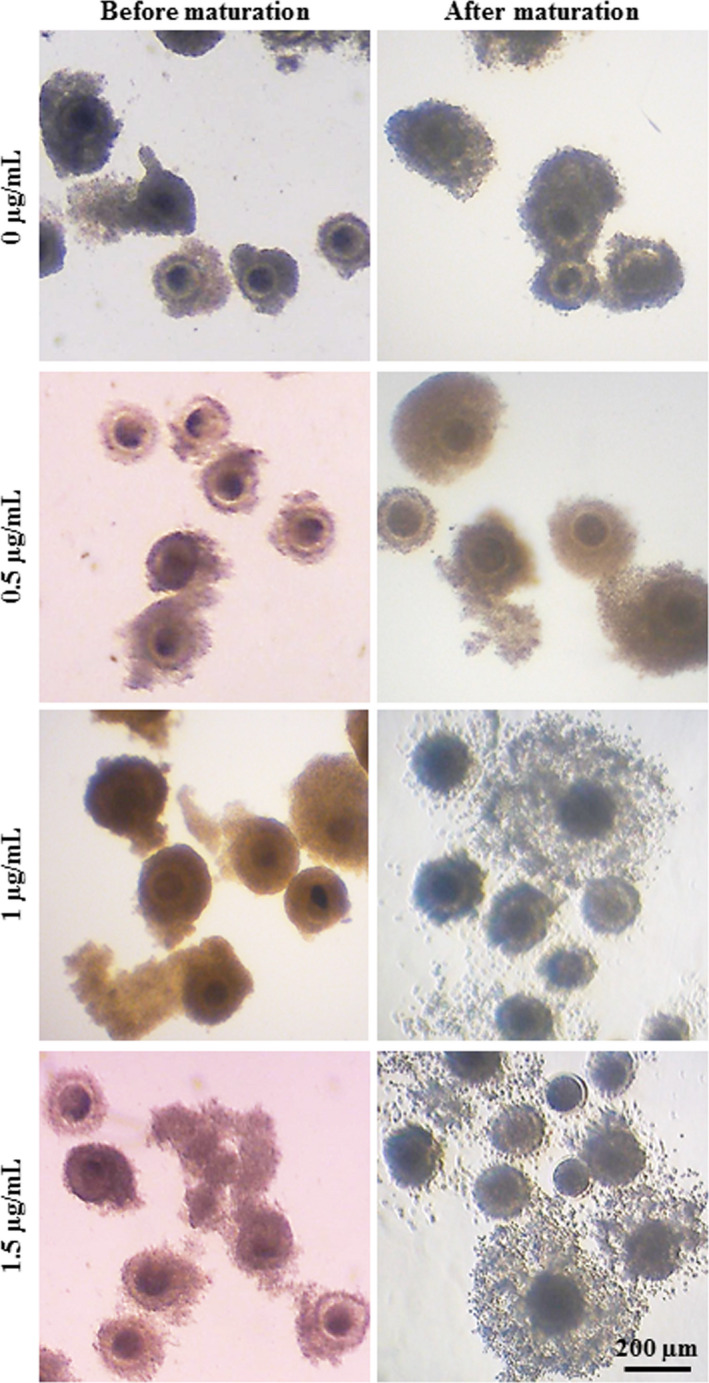
Goat cumulus oocyte complexes (COCs) before and after in vitro maturation. The in vitro maturation medium was supplemented with 0, 0.5, 1, and 1.5 µg/mL of 17β‐estradiol. Scale bar represents 200 μm

**FIGURE 3:**
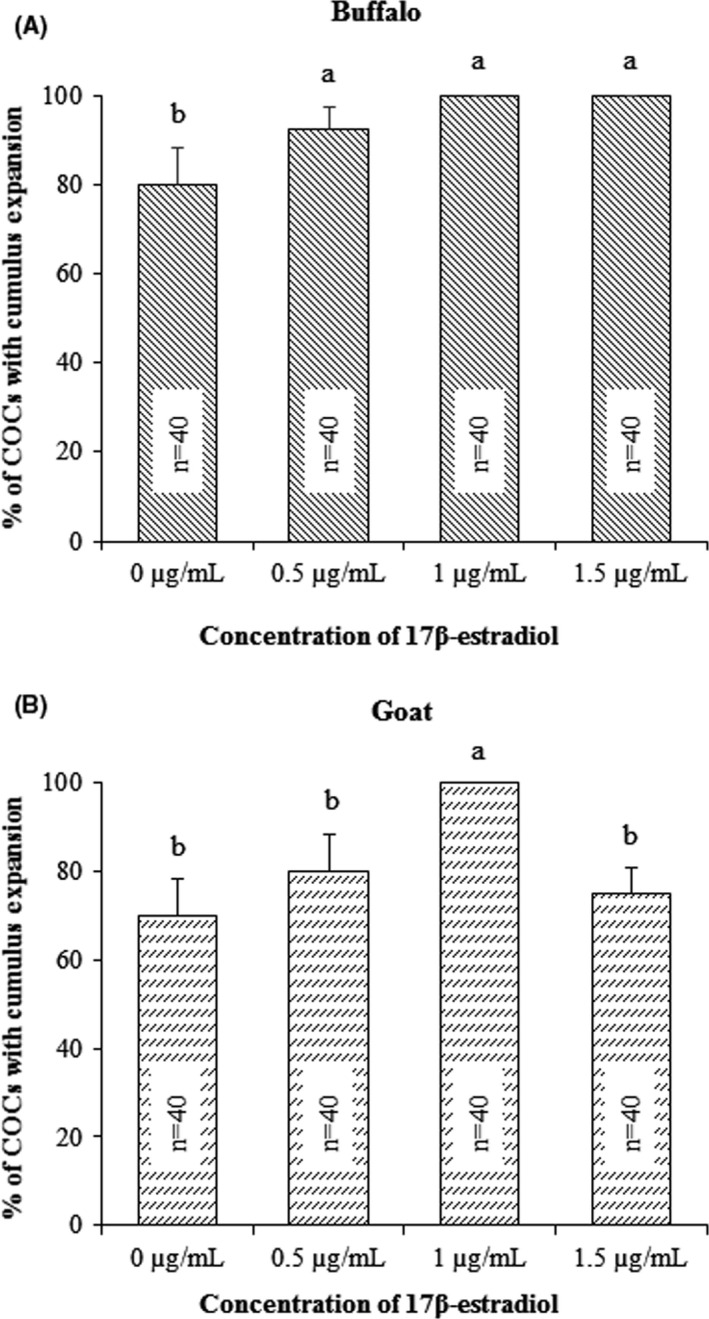
Effect of 17β‐estradiol on cumulus expansion of buffalo and goat COCs during in vitro maturation. Bar diagrams indicate the percentages of COCs with cumulus expansion after in vitro maturation of buffalo (A) and goat (B) oocytes. The numbers of complexes (n) used in each group are shown in each graph bar. Bars with different superscripts (a, b) differ significantly (*P* < .05)

The typical morphologies of COCs before and after in vitro maturation in the medium supplemented with 0, 0.5, 1, and 1.5 µg/mL of 17β‐estradiol in buffaloes and goats are shown in Figures 1 and 2, respectively. Assessment of oocyte maturation by visualization of cumulus expansion of COCs revealed a significant difference in expansion rate due to estradiol supplementation in both buffaloes and goats. In buffaloes, supplementation of in vitro maturation medium with 17β‐estradiol (0.5, 1.0 and 1.5 µg/mL) significantly increased cumulus expansion rate (93%, 100%, and 100%, respectively) compared to control (Figure 3A). In goats, supplementation of medium with 1 µg/mL 17β‐estradiol significantly increased cumulus expansion rate (100%) compared to 0, 0.5, and 1.5 µg/mL groups (73%, 80%, and 70%, respectively) (Figure 3B).

### 3.2 Effect of 17β‐estradiol on nuclear maturation of buffalo and goat oocytes

**FIGURE 4:**
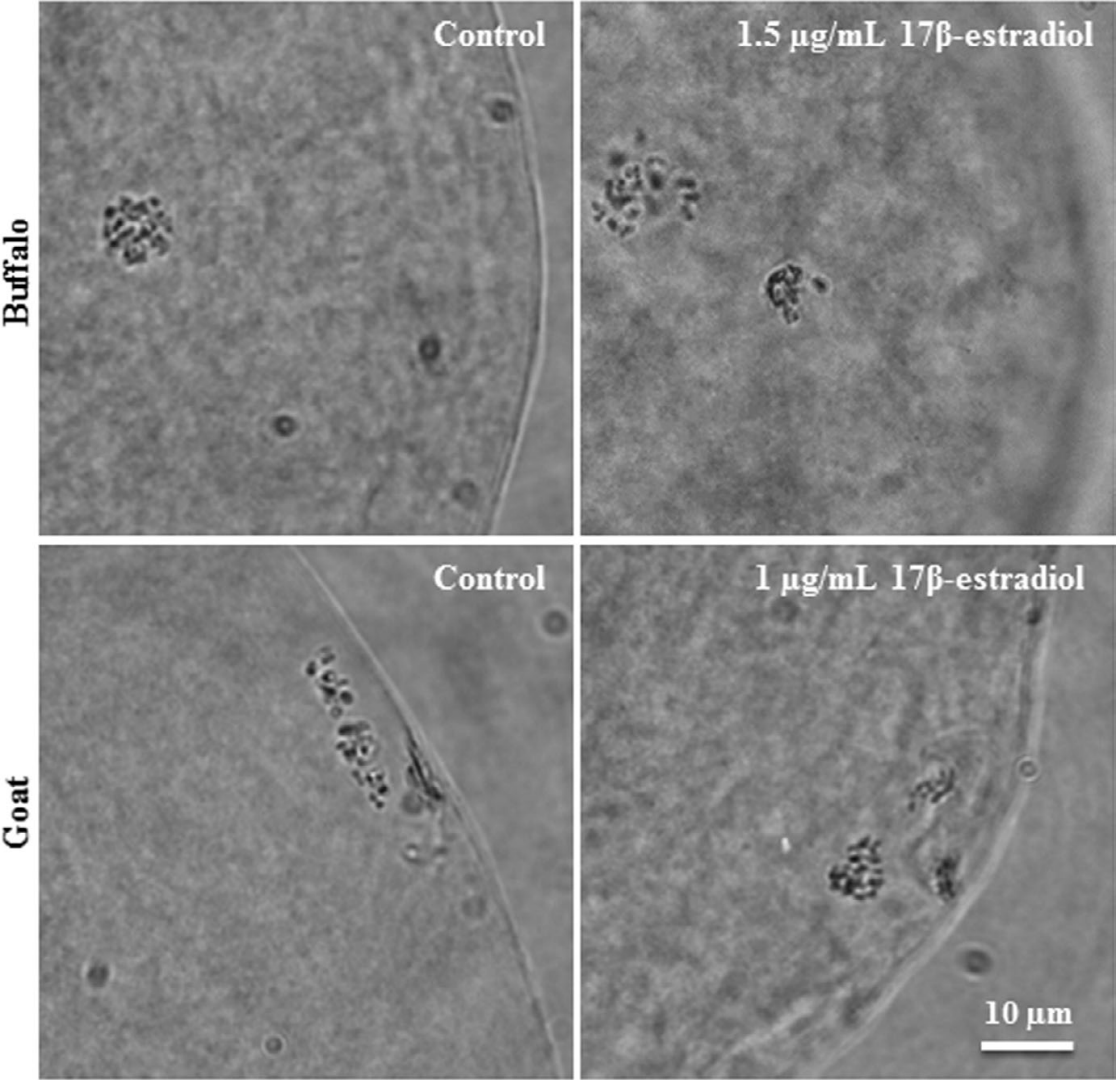
Nuclear morphologies of buffalo and goat oocytes after in vitro maturation. COCs were cultured in medium supplemented with 0, 0.5, 1, and 1.5 µg/mL of 17β‐estradiol during in vitro maturation. After maturation, oocytes were denuded mechanically, fixed with aceto‐ethanol, stained with aceto‐orcein, and examined for nuclear maturation. Scale bar represents 10 μm

**FIGURE 5:**
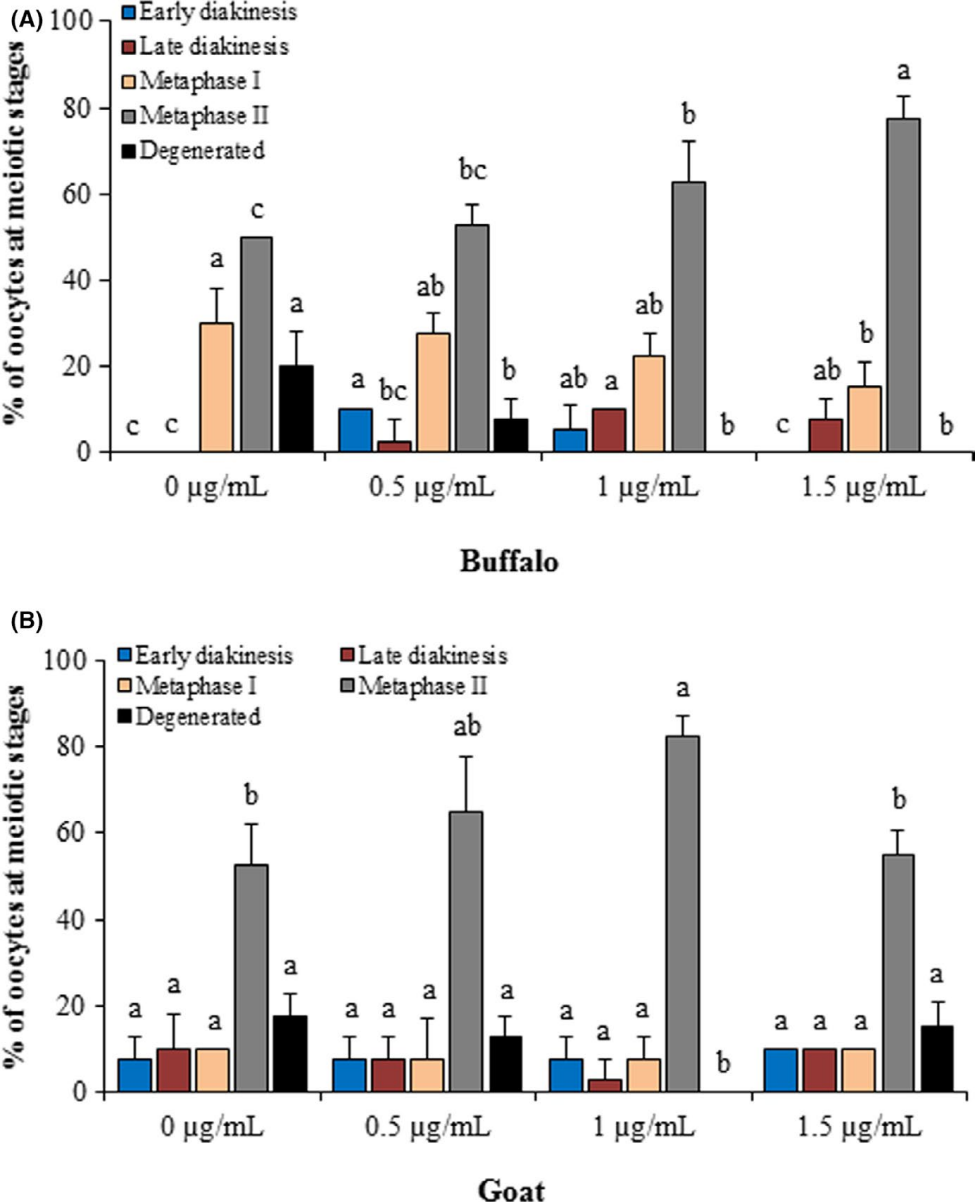
Effects of 17β‐estradiol on nuclear maturation of buffalo and goat oocytes. Vertical axis shows the percentages of oocytes at meiotic stages. The concentrations of 17β‐estradiol are shown in horizontal axis in buffalo (A) and goat (B)species, respectively. Bars with different color indicate different meiotic stages. Bars with different superscripts (a‐c) differ significantly (*P* < .05) in the same species

The representative images of nuclear morphology of buffalo and goat oocytes after maturation are shown in Figure 4. After resumption of meiosis, few proportion of buffalo oocytes were at early diakinesis in 0.5 µg/mL (10%) and 1 µg/mL (5%) of 17β‐estradiol supplemented groups (Figure 5A). The proportions of buffalo oocytes at late diakinesis increased with increment of 17β‐estradiol concentration. The percentages of MII oocytes increased in concentration‐dependent manner with 17β‐estradiol treatments.

In goat, the proportion of MII stage oocytes (83%) was higher in 1 µg/mL of 17β‐estradiol than 0 µg/mL (53%) and 1.5 µg/mL (55%) of 17β‐estradiol groups (Figure 5B). In addition, supplementation of medium with 1 µg/mL of 17β‐estradiol significantly reduced degenerated oocytes compared to the other treatments.

## 4 DISCUSSION

The rates of in vitro maturation of mammalian oocytes including mice, cattle, and sheep are low compared with oocytes matured in vivo.^28^, ^29^, ^30^ This might be due to poor nuclear maturation caused by inadequate culture conditions.^31^, ^32^, ^33^, ^34^ Several factors are included in culture media to improve the success rate of in vitro maturation. Here, we found that estradiol increased the percentage of MII oocytes. It is thought that estradiol supports nuclear maturation of oocytes during in vitro maturation. It has been reported that estradiol increases nuclear maturation of human oocytes.^35^ Steroid hormones act *via* two distinct mechanisms. The classical or genomic theory describes a model where intracellular nuclear receptors and transcriptional modulation of the genes are involved. Several reports have revealed the evidence against the classical mode of estradiol. The non‐genomic model of steroid action has been identified recently and many researchers have explained its characteristics.^36^, ^37^, ^38^ The non‐genomic steroid action does not involve transcriptional activation. Estradiol works on the cell surface and improves the potential of oocytes for development.^35^

For oocyte maturation, a bidirectional communication between oocytes and cumulus cells is essential.^39^ The morphology and metabolic activity of cumulus cells are changed during meiotic maturation of mammalian oocytes. Thus, cumulus cells influence maturation and developmental competence of oocytes.^40^, ^41^, ^42^, ^43^, ^44^, ^45^ Moreover, the cumulus cells synthesize the extracellular matrix. This phenomenon is known as cumulus expansion. The component in the expanded cumulus is hyaluronic acid (HA),^46^ and the amount of HA synthesis is correlated with the degree of expansion.^47^ The presence of oocytes is essential for FSH‐induced cumulus expansion.^48^, ^49^ It is also known that estradiol and oocyte‐derived paracrine factors including growth differentiation factor 9 (GDF9) and bone morphogenetic protein 15 (BMP15) promote the development and expansion of cumulus cells.^50^ Estradiol improves nuclear and cytoplasmic maturation as well as cumulus expansion in several species including bovine,^51^ equine,^52^ porcine,^53^ and caprine.^11^ Although estrogen supports the reproductive cycle in females, the necessity for estrogen in regulating specific events, including oocyte maturation, is species‐dependent.^20^ In the present study, supplementation of medium with 17β‐estradiol increased cumulus expansion rate in oocytes from both river buffaloes and Black Bengal goat oocytes. Thus, it is thought that 17β‐estradiol coordinates with oocyte‐derived factors in the culture medium, which in turn promotes cumulus expansion.

The concentration of estradiol for in vitro maturation of oocytes varies among different mammalian species. Concentration of 1 μg/mL is commonly used for oocyte maturation medium in the caprine,^54^, ^55^, ^56^ ovine,^57^, ^58^, ^59^ and bovine.^60^ This concentration mimics the presence of estradiol (1.1 ± 0.06 μg/mL) in preovulatory follicle in vivo.^61^ Higher concentrations (10 and 100 μg/mL) of estradiol prevented in vitro maturation of oocytes in boer goats.^18^ However, a clear comparison of effective concentrations of estradiol between different species has not been studied in the same culture condition. The present study compared the efficacy of estradiol on in vitro maturation of oocytes from Black Bengal goats and river buffaloes. Here, higher numbers of buffalo oocytes treated with 1.5 μg/mL of estradiol reached the MII stage than other groups. In goat, the number of oocytes at the MII stage significantly increased in 1 μg/mL estradiol but decreased with 1.5 μg/mL of estradiol, which further indicated that high concentration of estradiol inhibited meiotic maturation of goat oocytes. Previously, it was reported that a proportion of estradiol was absorbed by paraffin oil covering the microdrops of culture medium.^62^ In the present study, each droplet was placed in a separate culture dish and, thus, estradiol could not move from one droplet to another. It was considered that estradiol was equally absorbed from each droplet without affecting the estradiol concentration.

In conclusion, estradiol enhances nuclear maturation of oocytes in both buffaloes and goats.

## CONFLICT OF INTEREST

The authors declare that there is no conflict of interest that could be perceived as prejudicing the impartiality of the research reported.

## HUMAN/ANIMAL RIGHTS

This article does not contain any studies with human and animal subjects performed by the any of the authors.
